# Synchronous Breast Carcinoma, Uterine Myoma, and Ovarian Teratoma in a Single Woman

**DOI:** 10.7759/cureus.17977

**Published:** 2021-09-14

**Authors:** Saikat Das, Swagata Bramhachari, Ajay Halder, Ashwani Tandon, Ankit Lalchandani

**Affiliations:** 1 Radiotherapy, All India Institute of Medical Sciences Bhopal, Bhopal, IND; 2 General Surgery, All India Institute of Medical Sciences Bhopal, Bhopal, IND; 3 Obstetrics & Gynaecology, All India Institute of Medical Sciences Bhopal, Bhopal, IND; 4 Pathology and Laboratory Medicine, All India Institute of Medical Sciences Bhopal, Bhopal, IND

**Keywords:** breast cancer, synchronous tumour, multiple primary neoplasms, metachronous tumour, hereditary breast cancer, thyroid adenoma, ovarian teratoma, uterine leiomyoma

## Abstract

Multiple primary tumors in a patient diagnosed with invasive ductal breast cancer are rarely reported in the literature. Here we present a case of invasive ductal carcinoma of the breast in a 42-year-old lady, with synchronous uterine leiomyoma (UL), ovarian teratoma and with prior history of follicular adenoma of thyroid in the same patient. The clinical presentation and management plan is discussed with a review of the literature. Breast cancer is the most common cancer in women where the concomitant occurrence of multiple primary tumors is a diagnostic and therapeutic challenge. In low- and middle-income countries, where facilities of genetic screening in all patients of synchronous neoplasia are limited due to scarcity of resources, strong clinical suspicion, multidisciplinary management, and follow-up remain important.

## Introduction

Worldwide breast cancer is the most common malignancy among women with increased incidence in many low- and middle-income countries (LMIC) [1]. Breast cancer can be associated with multiple primary tumors synchronously (within six months) or metachronously in the same individual. The condition may be attributed to family history, immunologic and genetic defects, prolonged exposure to carcinogens, radiation and chemotherapy for primary cancer, and field cancerization [2]. Breast cancer is a hormone-dependent neoplasm related to increased estrogen (ER) exposure, with a risk of inherited genetic mutations like BRCA1 and 2, TP53, CHEK2, PTEN, CDH11, and STK11 [3]. Some hereditary breast cancer syndromes like Li-Fraumani syndrome, Cowden syndrome, Hereditary Breast and Ovarian Cancer syndrome, and Peutz-Jeghers syndrome are associated with multiple malignancies. Here we report synchronous breast invasive ductal carcinoma with ovarian and uterine neoplasms and metachronous thyroid adenoma, in a single patient. In LMIC, it is not possible to carry out genetic screening in all patients with synchronous neoplasia. Nevertheless, awareness and clinical suspicion especially among primary care physicians are important in such conditions. To the best of our knowledge, such multiple tumors in the same patient are rare, persuading us to report this condition along with the review of the literature.

## Case presentation

**Figure 1:**
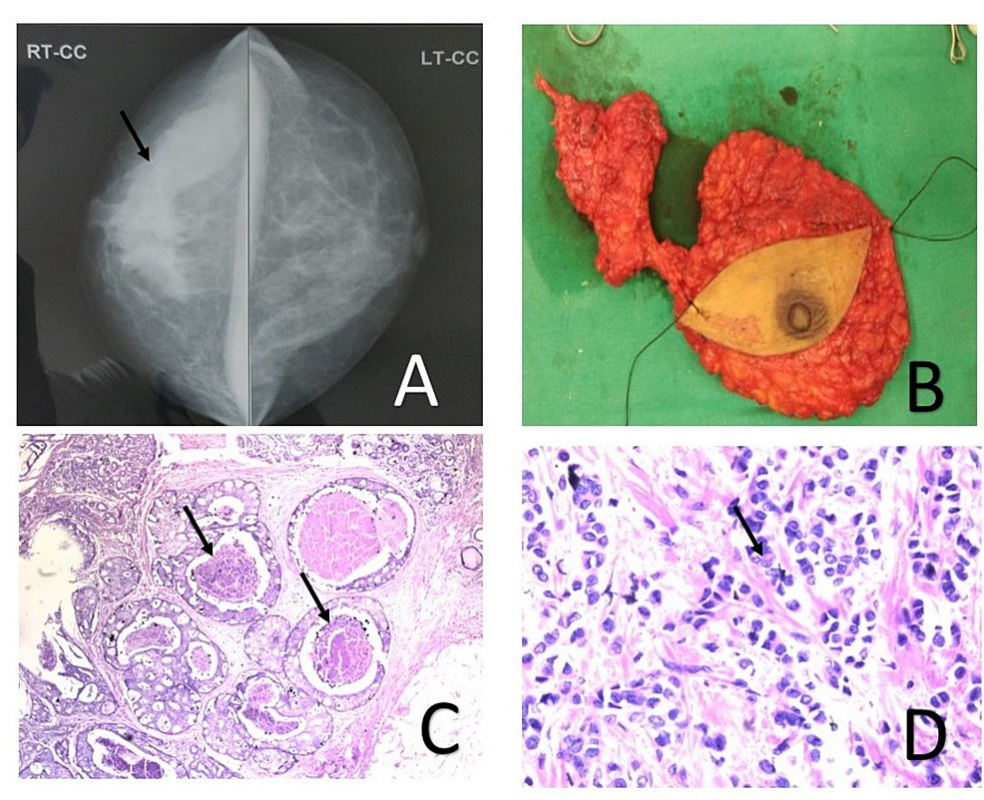
Breast carcinoma - (A) Mammography of right breast showing 4 cm × 3 cm BIRADS IV lesion in upper outer quadrant. (B) Gross view of surgical specimen of right modified radical mastectomy. (C) Histopathological section showing DCIS with comedo necrosis. (D) Histopathological section infiltrating duct carcinoma breast. DCIS, ductal carcinoma in situ

A 42-year-old premenopausal female, (gravida 2, para 2), presented with an eight-month history of a gradually increasing hard, non-tender lump (4 cm × 3 cm) in the upper outer quadrant of the right breast, without nipple discharge. The lump was not fixed to the overlying skin or underlying structures. Axillary lymph nodes were clinically not palpable. She had a history of menorrhagia for the one year before the presentation. On triple assessment, a BIRADS 4 lesion in the right breast was found on mammo-sonography (Figure 1A).

**Figure 2:**
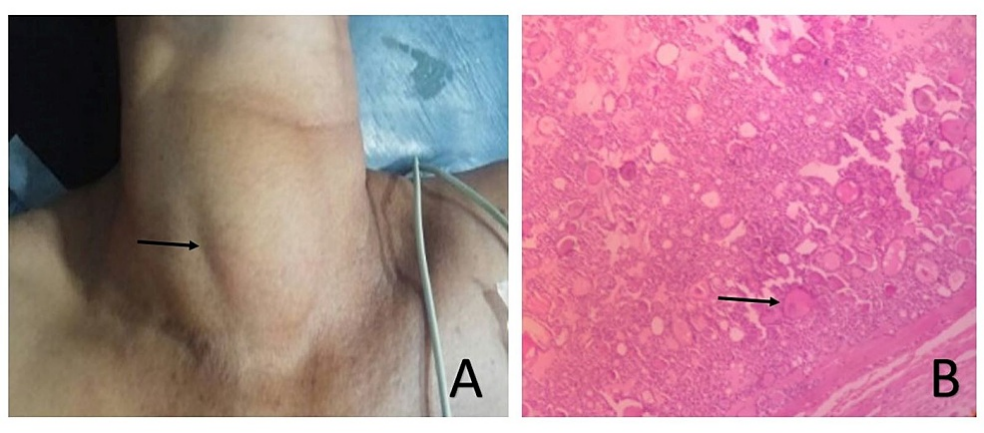
Thyroid adenoma. (A) Clinical picture showing goitre in the neck of size 7 cm × 4 cm. (B) Histopathological section showing closely packed follicles surrounded by fibrous capsule in follicular thyroid adenoma (H&E staining 10x). H&E, hematoxylin and eosin

Core biopsy suggested luminal A low infiltrating ductal carcinoma with associated ductal carcinoma in situ (DCIS). Abdomino-pelvic sonography revealed an intramural fibroid (7 cm × 8 cm × 8 cm), on the posterior uterine wall with a bilateral ovarian complex cyst. Four years back she underwent left hemithyroidectomy due to an increase in the size of a long-standing euthyroid goiter (>10 years). Histopathology was reported as follicular adenoma of the thyroid (Figure 2A,B).

She had no history of addiction to alcohol, loss of weight, bladder and bowel complaints, or any skin lesions. There is no history of any tumor or malignancy or history of multiple pathologies in the family. 

**Figure 3:**
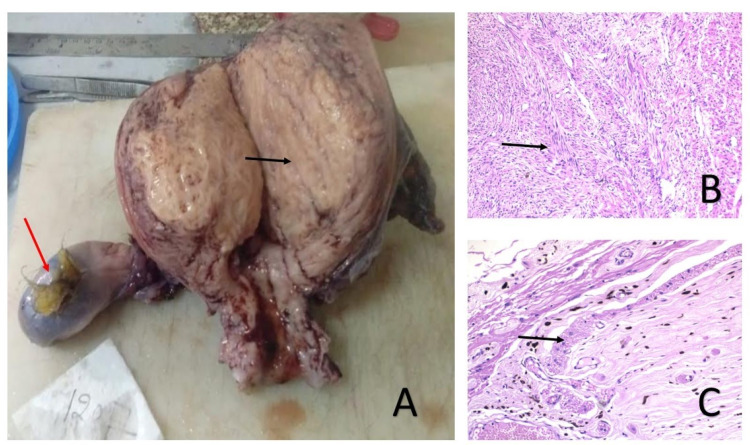
Reproductive tract neoplasm -- uterine myoma and bilateral ovarian tumor (teratoma). (A) Gross TAH+BSO specimen showing large intramural fibroid in the fundus, (black arrow) with bilateral ovarian cyst. Hairs are seen in left ovarian cyst suggestive of a dermoid cyst (teratoma) (red arrow). (B) Histopathological section of uterine myoma showing interlacing fascicles of spindle cells. (C) Histopathological section of right ovary showing ganglion cells containing neuromelanin. TAH+BSO, total abdominal hysterectomy + bilateral salpingo-oophorectomy

For the present complaint, she underwent a right modified radical mastectomy (Figure 1B). Postoperative histopathology confirmed infiltrating ductal carcinoma with extensive DCIS component without any lymph node metastasis, modified Bloom Richardson grade 2, (pT3N0M0) (Figure 1C,D). Receptor studies showed it as estrogen (ER) and progesterone (PR) positive but Her 2 neu negative. Four weeks later, she underwent a total abdominal hysterectomy with bilateral salpingo-oophorectomy. Per operatively, the presence of hair in the left ovarian cyst suggested a dermoid cyst. Histopathology of the retrieved specimen showed the presence of UL and bilateral mature ovarian teratoma (Figure 3A-C).

Tumor markers like cancer antigen (CA)-125 and carcinoembryonic antigen (CEA) were not elevated. Colonoscopy evaluation was normal. Chromosomal and genetic testing was not done due to financial constraints. The patient received adjuvant systemic chemotherapy and hormone therapy for breast carcinoma and is disease-free to date.

## Discussion

Billroth in 1889, first described the occurrence of multiple tumors in multiple organs of a single individual. Multiple primary tumors characterized by multiple tumors with different pathogenetic origins in the same patient are defined as more than one synchronous (within six months) or metachronous tumor in the same individual [2]. The only defined multiple tumor syndrome known to be associated with increased susceptibility to breast carcinoma and multiple malignancies in the same individual is Cowden syndrome. Patients with Cowden syndrome are at an increased risk of various malignancies like carcinoma breast, thyroid cancer, and uterine cancer but classical phosphatase and TENsin homolog deleted on chromosome 10 (PTEN) mutation features were not present in this case [4]. A multidisciplinary approach is required to optimally sequence the management of different tumors based on clinical priority. As the exact nature of the association between breast carcinoma and other organ neoplasm remains unknown, thorough history taking and careful clinical examination of patients is important. 

The observed relationship between genital tract neoplasm and breast cancer indicates that sex hormones might be the cause of common risk factors for this condition. Sex steroid hormonal pathways (in particular, estradiol) are associated with pathologies of the uterus and breast including endometrial cancer, endometriosis, UL, and breast cancer. A population-based study by Wise et al., in the USA, failed to find any association between the history of UL diagnosis and the overall incidence of breast cancer [5]. Two Asian studies based in Taiwan showed a higher incidence of breast cancer in patients with UL without increased mortality [6-7].

There are inconsistent results among studies on the association between benign ovarian tumors and breast cancer. These studies range from retrospective analysis to case-control design. A nationwide, registry-based study from Denmark by Gottschau et al., reported an increased risk of breast cancer in women with a benign solid ovarian tumor (standardized incidence ratio 1.09), persisting 20 years or more after the ovarian tumor diagnosis [8]. The exact association between ovarian teratoma and breast cancer has not been systematically studied though cases of Brenner tumor, struma ovarii associated with breast cancer has been reported in the literature [9-11].

There appears to be an increased concomitant incidence of benign thyroid diseases like thyroiditis and nodular goiter in breast cancer patients [12-13]. Increased incidence of secondary breast cancer in patients with thyroid cancer and vice versa have been reported in the literature [14]. Yu et al. reported a 1.65% incidence rate of breast cancer among patients with thyroid cancer which is significantly higher than the population breast cancer incidence rate of 0.27% in Chinese women [15]. This could be attributed to increased surveillance and close monitoring after diagnosis of the primary tumor. 

Ovarian teratomas and ULs are common tumors of the reproductive age group and may co-exist as ER stimulates the growth of both lesions [16-17]. Interestingly, Spinos et al. and Kim et al. observed an increased incidence of thyroid adenoma in patients with UL, suggesting that ER could be a common risk factor for causation and progression of both of these common benign tumours [18-19]. Therefore, the risk of breast carcinoma may be increased in patients with thyroid nodules, fibroid uterus, and ovarian teratomas due to ER stimulation [13, 16-19].

## Conclusions

From the perspective of clinical practice, in women with uterine myoma or thyroid nodule, clinical examination of the breast is essential to rule out any underlying lesions. Our case report suggests that women with uterine myoma and goiter should be aware of the potential risk of breast cancer development in their lifetime and so help in early diagnosis and management of breast carcinoma. Such a unique association of multiple neoplasms may not be causal, so knowledge of such associations between multiple tumors and syndromes, along with patient demographic and clinical manifestations can help to identify cancer predispositions. The gain of knowledge on patients with hereditary cancer and cancer survivors will hopefully allow the development of specific management and surveillance measures to improve the diagnosis and outcome of breast cancer.

The aim of this article is to report a rare occurrence of three benign and one malignant lesions in the same patient. Such association has been rarely reported in the literature and since this is a case report whether such occurrence is by chance or based on define association cannot be concluded which requires further multi-institutional and collaborative studies based on a larger number of patients. Since the occurrence of three or more tumors is rare, the aim of this case report is to provide literature that will encourage further clinical studies based on surveillance data/population.
